# The complete chloroplast genome of *Hydrangea strigosa* Rehder (Hydrangeaceae)

**DOI:** 10.1080/23802359.2021.1934145

**Published:** 2021-06-07

**Authors:** Jing Li, Zhi-Feng Fan, Chang-Le Ma

**Affiliations:** a School of Life Sciences, Southwest Forestry University, Kunming, China; b School of Landscape Architecture and Horticulture Sciences, Southwest Forestry University, Kunming, China; c Kunming University of Science and Technology, Kunming, China; d Southwest Landscape Architecture Engineering Research Center of National Forestry and Grassland Administration, Kunming, China

**Keywords:** *Hydrangea strigosa*, chloroplast genome, phylogenetic analysis

## Abstract

*Hydrangea strigosa* Rehder is a wild flowering shrub with high ornamental value. The complete chloroplast genome sequence of *H. strigosa* was characterized from Hiseq (Illumina Co., San Diego, CA) sequencing data. The chloroplast genome of *H. strigosa* is 157,905 bp in length with a pair of inverted repeats (IRs) (26,127 bp) which are separated by a large single-copy (LSC) (86,897 bp) and a small single-copy regions (SSC) (18,754 bp). It contains 131 genes, including 85 protein-coding genes, 38 tRNAs genes, and 8 rRNAs genes. The overall GC content of the whole genome is 37.80%. The maximum-likelihood phylogenetic analysis with the complete chloroplast genomes sequence of 22 species of Hydrangeaceae showed that *H. strigosa* is closely related to *H. davidii*.

*Hydrangea*, originates from East Asia, has a long history of use as an ornamental garden plant in temperate regions (Nashima et al. 2021; Yoshida et al. 2021). It is widely known that the color of *Hydrangea* sepal changes in the cultivating conditions (Yoshida et al. 2021), the sepal color is blue in acidic soil and red in alkaline soil. In addition, *Hydrangea* has strong ecological adaptability and heavy metal pollution resistance, it is also a hyperaccumulator of aluminum, which can be used as environmental remediation plant (Chen et al. 2020). Species circumscription and identification is notoriously difficult in the genus *Hydrangea* (Smet et al. 2017). Based on the ovary position, capsule tips and petal clutch characteristics, *Hydrangea* is divided into two sections, Sect. *Hydrangea* and Sect. *Calyptranthe*. The former can be divided into three series, namely *Petalanthae*, *Heteromallae,* and *Piptopetalae* (Cheng 1985). SRAP molecular markers showed that *Hydrangea strigosa* Rehder belonged to Ser. *Piptopetalae* (Chen and Peng 2012). *Hydrangea strigosa* is a 1–3 m tall deciduous shrub, and have a wide geographical distribution, dense to sparse forests or thickets in valleys, trail sides on mountain slopes (Wei 1995; Smet et al. 2017). Because of its large inflorescence, *H. strigosa* is a wild flowering shrub with high ornamental value. The study for *H. strigosa* has been focused on seedling technique and physiological characteristics (Chen et al. 2020; Li et al. 2020), there was no record of complete chloroplast genome sequence to date. In this study, we characterized a complete chloroplast genome of *H. strigosa* and confirmed the phylogenetic relationship of the genus, to provide genetic information for further research on phylogeography, genetic diversity and evolution.

Fresh leaves of *H. strigosa* were collected from Baijixun Township, Weixi County, Diqing Tibetan Autonomous Prefecture, Yunnan Province, China (99°1′32.67″E, 27°29′30.1″N). Voucher specimen (SWFU20200713MFY) was deposited in the Herbarium of Southwest Forestry University, China. Total genomic DNA was extracted from silica gel dried leaf tissues using a modified CTAB method (Doyle and Doyle 1987). Short-insert library (insert size, 300 bp) was prepared and then sequenced using the Illumina HiSeq 2500-PE150 platform (Illumina, San Diego, CA). The clean reads was obtained from filtered raw reads using NGS QC Toolkit version 2.3.3 with default parameters (Patel and Jain 2012). The plastome was de novo assembled by NOVOPlasty (Dierckxsens et al. 2017) and annotated by Geneious 20.0.3 (Kearse et al., 2012) with the complete chloroplast genome sequence of *H. davidii* (NC_050783) as the reference genome. The complete chloroplast genome was submitted to GenBank with Accession no. MW218933.

The complete chloroplast genome of *H. strigosa* is 157,905 bp in length with a quadripartite structure, including a large single-copy region of 86,897 bp, a small single-copy region of 18,754 bp, and two inverted repeat (IR) regions of 26,127 bp. The overall GC content is 37.80%, and the corresponding values of the LSC, SSC, and IR regions are 36.0%, 31.7%, and 43.1%, respectively. The genome encodes 131 genes, including 85 protein-coding genes (PCGs), 38 transfer RNA genes (tRNAs), and 8 ribosomal RNA genes (rRNAs). A total of 54 SSRs were discovered by the online software MISA-web (Beier et al. 2017). Among them, the numbers of mono-, di-, tri-, tetra- and penta-nucleotides SSRs are 45, 2, 2, 3, and 2, respectively.

To determine the phylogenetic location of *H. strigosa*, we used the complete chloroplast genomes sequence of 22 species of Hydrangeaceae including 13 *Hydrangea* species to construct phylogenetic tree. All cp genome sequences were aligned using MAFFT (Katoh and Standley 2013) with default parameters, and then the maximum likelihood tree was constructed using RAxML version 8.2.11 (Stamatakis 2014) in which the GTR + G DNA substitution model was selected as a best-fit model using PartitionFinder2 (Lanfear et al. 2016), all branch nodes were calculated under 1000 bootstrap replicates. The phylogenetic analysis revealed that all species of *Hydrangea* were formed one monophyletic clade. Phylogenetic tree showed that *H. strigosa* was sister to *H. davidii* with strongly supported under current sampling (Figure 1).

**Figure 1.**
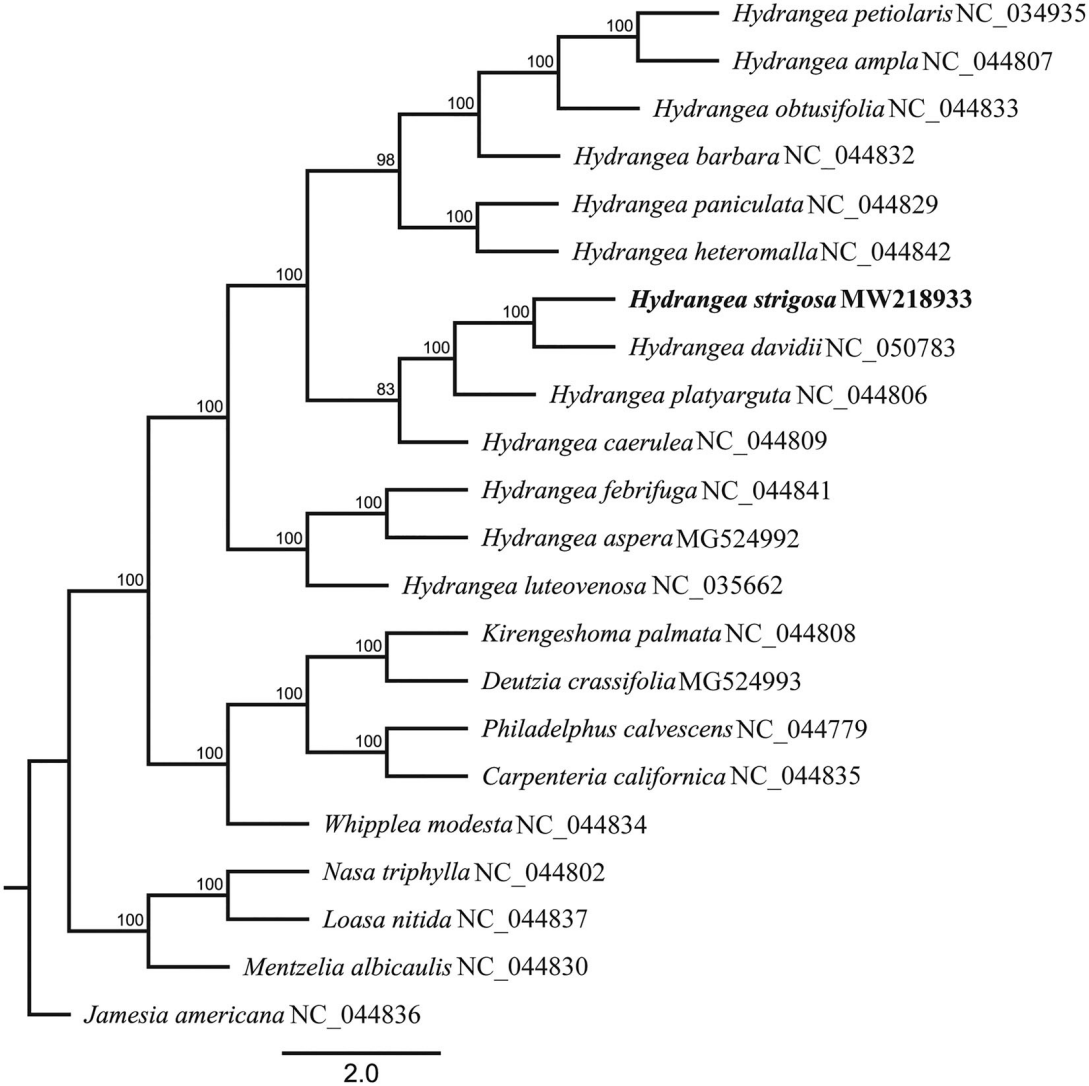
Maximum-likelihood phylogenetic tree reconstructed by RAxML based on complete chloroplast genome sequences from 13 *Hydrangea* species and 9 other species of Hydrangeaceae. Numbers on branches are bootstrap support values.

## Data Availability

The data that support the findings of this study are openly available in GenBank at https://www.ncbi.nlm.nih.gov/, reference number [MW218933] [SRR12989400], or obtain from the corresponding author.
